# From Barriers to Action: Operationalizing Implementation Strategies for the GOC Video in Nursing Homes

**DOI:** 10.1093/geroni/igaf122.3940

**Published:** 2025-12-31

**Authors:** Latarsha Chisholm, Susan Hickman, Kathleen Unroe

**Affiliations:** University of Central Florida, Florida, United States; Indiana University, Indianapolis, Indiana, United States; Indiana University School of Medicine, Indianapolis, Indiana, United States

## Abstract

Implementation challenges have limited the widespread adoption of evidence-based strategies to improve advance care planning for nursing home residents and their families. Implementation strategies are critical for influencing outcomes and enhancing the adoption of evidence-based interventions. This study explored the use of implementation strategies to support the Goals of Care (GOC) video—a 20-minute decision aid designed to help family caregivers of persons living with dementia make healthcare decisions aligned with their values. The GOC video is part of a broader intervention that includes staff training and structured care plan meetings. Semi-structured group interviews were conducted with 12 staff members from six Florida nursing homes. Content analysis identified key barriers to implementation, and the CFIR-ERIC matching tool was used to align appropriate strategies to address them. Common barriers included the video’s length, limited access to understandable health education, caregiver readiness, and cultural or family background differences. Recommended implementation strategies included promoting adaptation, distributing diverse educational materials, assessing readiness, identifying barriers and facilitators, and fostering learning collaboratives. Action staff can take include adapting the video to local contexts while preserving core components and integrating it into Quality Assurance & Performance Improvement (QAPI) programs to promote advance care planning. Action the research team can take includes supporting implementation by co-developing culturally appropriate materials, conducting pre-implementation readiness assessments, identifying enablers and barriers, and sharing best practices across sites. This study highlights key barriers and actionable strategies for implementing the GOC video in nursing home settings.

